# When Acarbose Overdose Turns Severe: Managing Hypoglycemia in an Uncommon Scenario

**DOI:** 10.1002/ccr3.71448

**Published:** 2025-11-11

**Authors:** Aron Shrestha, Navin Poudel, Kshitiz Acharya, Arjun Chaudhary, Manish Acharya, Santosh Bastola, Bibek Ghimire

**Affiliations:** ^1^ Maharajgunj Medical Campus Tribhuvan University Teaching Hospital Kathmandu Nepal; ^2^ Enam Medical College Dhaka University Dhaka Bangladesh; ^3^ Department of Emergency Medicine Tribhuvan University Teaching Hospital Kathmandu Nepal; ^4^ Gandaki Medical College Tribhuvan University Kathmandu Nepal

**Keywords:** acarbose, drug overdose, emergency management, hypoglycemia

## Abstract

Acarbose is usually safe, but overdose can trigger severe, life‐threatening hypoglycemia. In cases of unexplained low blood sugar—particularly in patients with psychiatric illness or access to hypoglycemic drugs—acarbose toxicity should be considered. Rapid intravenous glucose, careful monitoring, and patient education are vital for optimal outcomes.

## 1 Introduction

Alpha‐glucosidase inhibitors (AGIs), like acarbose, are oral medications commonly used to manage blood sugar levels in diabetic patients [1]. Derived from the fermentation of the microorganism *Actinoplanes*, acarbose works by blocking enzymes in the intestines that break down carbohydrates [2]. It inhibits α‐glucosidase enzymes in the gut in a competitive and reversible manner, slowing carbohydrate digestion and absorption, thereby lowering blood sugar after meals [3]. Some studies have confirmed that acarbose helps reduce post‐meal blood sugar levels in patients with abnormal glucose regulation. The comparatively higher dietary starch intake observed in Asian populations also contributes to a more pronounced hypoglycemic response in these groups [4].

Hypoglycemia can be diagnosed in the presence or absence of symptoms if the plasma glucose concentration is 50 mg per dL or less, and may be caused by numerous drugs [5]. Intentional overdose is more common among diabetic patients with concomitant psychiatric illness [6]. In cases of unexplained hypoglycemia in nondiabetic individuals, especially those with access to hypoglycemic drugs, surreptitious ingestion or suicide attempts should be considered [7].

In cases of massive acarbose overdose, elevations in hepatic transaminases have been reported, potentially indicating a risk of liver injury. This underscores the importance of baseline liver function assessment during the diagnostic evaluation [6]. Initial management of acarbose overdose includes withholding foods or beverages containing carbohydrates for 4–6 h. Severe hypoglycemia may require intravenous glucose or intramuscular glucagon administration [8].

We herein report the first documented case of intentional acarbose overdose in an Asian patient, which was successfully managed through timely intervention.

## 2 Case History/Examination

A 24‐year‐old male presented to our emergency department with a significant decrease in consciousness and profuse sweating following the intentional ingestion of 21 tablets of 25 mg Acarbose, totaling a dose of 525 mg. The circumstances suggested a possible intentional overdose, and he was admitted for further evaluation and management. The patient had no prior diagnosis of diabetes mellitus or any other chronic medical illness. However, he had a known history of anxiety disorder, for which he had been receiving pharmacological treatment intermittently. There was no prior history of psychiatric hospitalization.

He presented with a Glasgow Coma Scale (GCS) score of 8/15. His vital signs were notable for a pulse rate of 68 beats per minute, blood pressure of 80/40 mmHg, oxygen saturation (SpO_2_) of 96% on room air, and a body temperature of 97°F. Despite receiving prompt initial intervention, the patient declined hospital admission and left against medical advice after his first one‐day stay. However, over the following hours, he developed worsening symptoms characterized by prolonged episodes of reduced consciousness, profuse diaphoresis, and lethargy. Due to the persistence and severity of these hypoglycemic episodes, he was readmitted for a second time, 2 days after his initial discharge, and remained hospitalized for 2 days. On readmission, his GCS had improved to 14/15. His vital signs at that time were stable, including a respiratory rate of 18 breaths per minute, oxygen saturation of 100% on room air, pulse rate of 60 beats per minute, blood pressure of 100/80 mmHg, and a body temperature of 97.2°F, with no evidence of hypothermia. The patient's height was 170 cm and his weight was approximately 68 kg, corresponding to a BMI of 23.5 kg/m^2^, which is within the normal range based on World Health Organization criteria.

On general examination, the patient was unconscious. Pupils were equal and reactive to light, with no focal neurological deficits. There were no signs of liver disease or adrenal insufficiency. Other examinations were also normal. Due to the persistence and severity of these hypoglycemic episodes, he was subsequently readmitted for further management.

## 3 Investigations, Diagnosis, and Treatment

Initial laboratory investigations were concerning, revealing a critically low blood glucose level of 22 mg/dL (89–104 mg/dL). Immediate management included two boluses of 50% Dextrose, 50 mL IV each (totaling 100 mL or 50 g of glucose), administered STAT to rapidly counteract the critically low blood sugar. This was followed by a continuous infusion of Dextrose Normal Saline (DNS) 1 L IV over 20 min, providing an additional 50 g of glucose, along with Normal Saline (NS) 1 L IV over 30 min for hydration. Following these interventions, the patient's blood glucose levels were closely monitored every 2 h. The effectiveness of the treatment was confirmed by subsequent laboratory analysis, which revealed a normal random glucose level of 5.7 mmol/L (within the reference range of 3.8–7.8 mmol/L), indicating successful resolution of the acute hypoglycemic episode. The patient was also started on Esofest to manage potential gastrointestinal complications.

An upper abdominal ultrasound was performed to assess for any potential internal injuries or complications. The scan indicated that the liver, gallbladder, spleen, and common bile duct (CBD) were normal. However, a mild dilatation of the left renal pelvis with pyelectasis was observed, warranting further monitoring.

As shown in Table 1, additional laboratory tests showed normal levels of gamma‐glutamyl transferase (Gamma GT) and albumin, and troponin I was negative, ruling out acute cardiac injury. Serological tests were all non‐reactive. Despite these normal findings, the patient's total leukocyte count (TLC) was elevated, indicating an ongoing stress response or potential infection. Liver function tests (LFT) were within normal limits, but urea and creatinine levels were elevated, indicating mild renal impairment likely secondary to the acarbose overdose. Interestingly, despite the critical hypoglycemia noted on arrival, subsequent random glucose levels returned to within normal limits following treatment. The urine routine examination (RE) showed no abnormalities, ruling out urinary tract infections or other renal pathologies as a contributing factor to his presentation.

**TABLE 1:** Key laboratory parameters in a hypoglycemic patient.

Category	Test	Result	Unit	Reference range
Liver function	Total Bilirubin	9	μmol/L	Adults: 5–21
	Direct Bilirubin	2	μmol/L	< 4
	SGPT/ALT	19	U/L	0–50
	SGOT/AST	40	U/L	0–50
	Alk Phosphatase	57	U/L	30–120
	Albumin	41	gm/L	Adults: 35–52
	Gamma GT	21	U/L	0–55
Hematology (CBC)	TLC (Total Leukocyte Count)	14,700	/cmm	4000–11,000
	Neutrophils	88	%	45–75
	Lymphocytes	10	%	25–45
	Hb (Hemoglobin)	16.5	gm%	12–18
Kidney function	Urea	1.3	mmol/L	Adults: 2.8–7.2
	Creatinine	57	μmol/L	Female: 58–96; Male: 72–127
	Sodium	136	mEq/L	135–146
	Potassium	4	mEq/L	3.5–5.2

The patient received continuous intravenous fluids and glucose supplementation to manage the recurring hypoglycemic episodes. He was closely monitored in the emergency department for any further complications related to the acarbose overdose and the hypoglycemic spells.

## 4 Outcome and Follow‐Up

A 1 month follow‐up ensured positive progress in counseling sessions, with the patient expressing a better understanding of medication safety and demonstrating improved coping skills for stress. The patient's blood glucose levels, monitored daily, remained stable, with no hypoglycemic episodes noted. HbA1c results indicated good glycemic control. The patient showed good adherence to dietary recommendations and medication instructions.

## 5 Discussion

Reports of acarbose overdose are rare in the literature. To our knowledge, this is the first documented account of acarbose overdose with successful management. Clinical and surveillance studies have revealed that acarbose treatment is associated with very few serious side effects [9], and its tolerability appears independent of age [10].

AGIs like acarbose help manage type 2 diabetes by delaying carbohydrate digestion, which may lead to gastrointestinal side effects such as flatulence and diarrhea due to the fermentation of undigested carbohydrates in the colon. It carries a risk of potentially severe hepatotoxicity, so regular liver function tests are advised—every 3 months during the initial year and periodically thereafter. It also interacts with various drugs, such as thiazides, steroids, warfarin, and chlorpromazine [11]. To minimize these effects, it's recommended to start with a low dose and gradually increase it to find the effective dose for glycemic control [12]. For patients experiencing gastrointestinal symptoms, dosage reduction may be necessary [13].

In overdose situations, excessive acarbose can inhibit carbohydrate digestion significantly, resulting in severe hypoglycemia, especially if the patient has not ingested sufficient glucose [8]. Therapeutically, the mean dose is three 100 mg tablets daily [14]. In this case, the patient ingested 525 mg of tablet intentionally, leading to severe hypoglycemia with symptoms including decreased consciousness and sweating, confirmed by a glucose level of 22 mg/dL. These symptoms are critical conditions requiring immediate medical attention. His respiratory rate and SpO_2_ were stable, though his pulse rate was reduced, possibly due to hypoglycemia.

In a controlled clinical trial, doses as high as 600–900 mg have been associated with gastrointestinal adverse effects and high dropout rates due to carbohydrate malabsorption [10]. The diagnosis of hypoglycemia due to acarbose overdose was supported by the patient's history of ingestion, clinical presentation, and laboratory findings. The confirmation came from the low blood glucose levels. It is crucial to consider the possibility of other causes of hypoglycemia in the differential diagnosis, such as other drug overdoses, insulinoma, or adrenal insufficiency, but the context of a known acarbose overdose makes it the most likely cause in this case [15].

Treatment for hypoglycemia typically involves fast‐acting carbohydrates if the patient is alert or intravenous dextrose or glucagon if the patient is unresponsive or cannot take food orally [16]. Here, a 50% dextrose solution was used to rapidly increase blood glucose levels, with DNS for ongoing glucose maintenance. Continuous monitoring is essential to avoid rebound hyperglycemia, which could lead to endogenous insulin release and recurrent hypoglycemia [17].

Patients with drug‐induced hypoglycemia generally have a good prognosis if the condition is recognized early and treated promptly. However, the patient's condition can complicate the prognosis as ongoing monitoring and management are crucial. Education on the risks of hypoglycemia and the importance of follow‐up care should be emphasized. In our case, a one‐month follow‐up of the patient was ensured well with stable vital signs and blood glucose level under control.

## 6 Conclusion

In conclusion, this case highlights the critical nature of acarbose overdose, leading to severe hypoglycemia. Rapid diagnosis and intervention with intravenous glucose were crucial in stabilizing the patient and preventing further complications. It is effective, safe, and well tolerated when taken in a therapeutic dose.

## Author Contributions

**Aron Shrestha:** conceptualization, writing – original draft, writing – review and editing. **Navin Poudel:** conceptualization, visualization, writing – original draft. **Kshitiz Acharya:** conceptualization, writing – original draft. **Arjun Chaudhary:** writing – review and editing. **Manish Acharya:** conceptualization, supervision. **Santosh Bastola:** conceptualization, visualization. **Bibek Ghimire:** writing – review and editing.

## Disclosure

Declaration: All the authors declare that the information provided here is accurate to the best of our knowledge.

## Ethics Statement

Since this is a case report, our Institutional Review Board of the Institute of Medicine, Tribhuvan University has waived the requirement for ethical approval.

## Consent

Written informed consent was obtained from the patient for publication and any accompanying images.

## Conflicts of Interest

The authors declare no conflicts of interest.

## Data Availability

The datasets used during this study will be available from the corresponding author upon reasonable request.
